# Supplementary material for: Structure and predictors of in-hospital nursing care leading to reduction in early readmission among patients with schizophrenia in Japan: A cross-sectional study
Source: PLoS One. 2021 Apr 30;16(4):e0250771. doi: 10.1371/journal.pone.0250771 (PMC8087037; doi:10.1371/journal.pone.0250771)
Supplement: S1 Fig — (DOCX) [file pone.0250771.s002.docx]

**S1 Fig. In-hospital nursing care leading to reduction in early readmission**
