# Supplementary material for: Structure and predictors of in-hospital nursing care leading to reduction in early readmission among patients with schizophrenia in Japan: A cross-sectional study
Source: PLoS One. 2021 Apr 30;16(4):e0250771. doi: 10.1371/journal.pone.0250771 (PMC8087037; doi:10.1371/journal.pone.0250771)
Supplement: S1 Table — (DOCX) [file pone.0250771.s003.docx]

**S1 Table. Descriptive statistics of the IRERSS (n = 724)**

| **Total: IRERSS**  median = 132, mean = 131.92 (SD = 18.09) | | n  (%) | | | | | mean  (SD) |
| --- | --- | --- | --- | --- | --- | --- | --- |
|  |  | 1 | 2 | 3 | 4 | 5 |  |
| **Factor 1: Promoting cognitive functioning and self-care** | | | | | median = 32 | | 32.31  (5.29) |
| #30 | I talked with the patient about how to deal with delusions so that he/she could take responsibility for behaviors. | 3  (.4) | 56  (7.7) | 291  (40.2) | 316  (43.6) | 58  (8.0) | 3.51  (.768) |
| #29 | I helped the patient reconsider his/her thoughts so that he/she could realize that his/her delusions were thoughts that were inconsistent with reality. | 5  (.7) | 51  (7.0) | 299  (41.3) | 311  (43.0) | 58  (8.0) | 3.51  (.770) |
| #33 | I observed how the patient deals with delusions. | 5  (.7) | 52  (7.2) | 321  (44.3) | 286  (39.5) | 60  (8.3) | 3.48  (.775) |
| #31 | I evaluated my nursing care from changes in the patient’s behaviors. | 3  (.4) | 67  (9.3) | 300  (41.4) | 286  (39.5) | 68  (9.4) | 3.48  (.805) |
| #32 | I believed in the patient and encourage him/her to change behaviors. | 3  (.4) | 57  (7.9) | 270  (37.3) | 324  (44.8) | 70  (97) | 3.55  (.790) |
| #34 | I helped the patient understand the need for medication by providing factual information. | 4  (.6) | 23  (3.2) | 237  (32.7) | 353  (48.8) | 107  (14.8) | 3.74  (.765) |
| #35 | I tried to notice changes in the patient’s attitudes toward medication | 5  (.7) | 32  (4.4) | 238  (32.9) | 348  (48.1) | 101  (14.0) | 3.70  (.7879 |
| #28 | I helped the patient improve his/her lifestyle. | 2  (.3) | 33  (4.6) | 237  (32.7) | 369  (51.0) | 83  (11.5) | 3.69  (.743) |
| #26 | I helped the patient accept his/her disability. | 3  (.4) | 38  (5.2) | 242  (33.4) | 363  (50.1) | 78  (10.8) | 3.66  (.756) |
| **Factor 2: Identifying reasons for readmission** | | | | | median = 31 | | 30.35  (4.48) |
| #9 | I tried to understand the patient’s capabilities and the challenges that he/she had been facing. | 3  (.4) | 31  (4.3) | 209  (28.9) | 382  (52.8) | 99  (13.7) | 3.75  (.756) |
| #8 | I tried to gain a more detailed understanding about why the patient was readmitted. | 4  (.6) | 45  (6.2) | 255  (35.2) | 324  (44.8) | 96  (13.3) | 3.64  (.809) |
| #7 | I tried to understand the challenges that the patient had been facing based on my observations of his/her daily life. | 3  (.4) | 29  (4.0) | 245  (33.8) | 366  (50.6) | 81  (11.2) | 3.68  (.739) |
| #6 | As a nurse, I paid close attention to the things about which the patient was worried. | 4  (.6) | 17  (2.3) | 193  (26.7) | 415  (57.3) | 95  (13.1) | 3.80  (.709) |
| #10 | I envisioned nursing goals that aimed to enhance the post-discharge well-being of the patient. | 3  (.4) | 21  (2.9) | 179  (24.7) | 395  (54.6) | 126  (17.4) | 3.86  (.745) |
| #11 | I created nursing plans that incorporated the opinions of other nursing staff members. | 4  (.6) | 23  (3.2) | 171  (23.6) | 405  (55.9) | 121  (16.7) | 3.85  (.747) |
| #5 | I shared important information about the patient with other nursing staff members. | 4  (.6) | 20  (2.8) | 141  (19.5) | 430  (59.4) | 129  (17.8) | 3.91  (.725) |
| #4 | I often cooperated with other nursing staff members. | 4  (.6) | 3  (4.6) | 168  (23.2) | 373  (51.5) | 146  (20.2) | 3.86  (.805) |
| **Factor 3: Establishing cooperative systems within the community** | | | | | median = 25 | | 24.66  (4.89) |
| #42 | I discussed with the patient the services that he/she wanted to use in his/her community. | 28  (3.9) | 68  (9.4) | 246  (34.0) | 293  (40.5) | 89  (12.3) | 3.48  (.957) |
| #40 | I participated in care conferences that involved community care providers. | 35  (4.8) | 66  (9.1) | 229  (31.6) | 271  (37.4) | 123  (17.0) | 3.53  (1.031) |
| #43 | I informed the patient and his/her family about the support system that would be available to the patient after discharge. | 18  (2.5) | 62  (8.6) | 252  (34.8) | 294  (40.6) | 98  (13.5) | 3.54  (.917) |
| #41 | I assessed the patient’s self-care abilities by comparing his/her behaviors at the time of admission and discharge. | 23  (3.2) | 66  (9.1) | 289  (39.9) | 270  (37.3) | 76  (10.5) | 3.43  (.910) |
| #38 | I put the patient in touch with community nurses to ensure the continuity of care. | 13  (1.8) | 66  (9.1) | 251  (34.7) | 300  (41.4) | 94  (13.0) | 3.55  (.894) |
| #37 | I was in touch with the patient and his/her caregivers, for a while, even after discharge. | 9  (1.2) | 56  (7.7) | 266  (36.7) | 318  (43.9) | 75  (10.4) | 3.54  (.828) |
| #39 | I discussed with the patient the good things that could happen to him/her after discharge with him/her. | 6  (.8) | 60  (8.3) | 245  (33.8) | 324  (44.8) | 89  (12.3) | 3.59  (.839) |
| **Factor 4: Sharing goals about community life** | | | | | median = 26 | | 25.70  (4.19) |
| #15 | I discussed with the patient and his/her family how he/she can adapt to community life. | 2  (.3) | 40  (5.5) | 199  (27.5) | 354  (48.9) | 129  (17.8) | 3.78  (.808) |
| #14 | I discussed with the patient and his/her family what he/she wanted to do in his/her community. | 2  (.3) | 49  (6.8) | 252  (34.8) | 337  (46.5) | 84  (11.6) | 3.62  (.787) |
| #17 | I helped the patient prepare for community life. | 2  (.3) | 47  (6.5) | 238  (32.9) | 369  (51.0) | 68  (9.4) | 3.63  (.754) |
| #16 | I helped the patient practice what he/she was not good at. | 2  (.3) | 36  (5.0) | 207  (28.6) | 387  (53.5) | 92  (12.7) | 3.73  (.753) |
| #18 | I reassured the patient and his/her family about his/her ability to adapt to community life after discharge. | 1  (.1) | 28  (3.9) | 241  (33.3) | 377  (52.1) | 77  (10.6) | 3.69  (.716) |
| #13 | I relieved the anxiety that the patient and his/her family experienced about community life. | 3  (.4) | 72  (9.9) | 288  (39.8) | 296  (40.9) | 65  (9.0) | 3.48  (.809) |
| #12 | I valued the happiness of the patient and his/her family. | 3  (.4) | 28  (.9) | 216  (29.8) | 372  (51.4) | 105  (14.5) | 3.76  (.760) |
| **Factor 5: Creating restful spaces** | | | | | median = 19 | | 18.89  (2.71) |
| #23 | I created spaces within which the patient did not feel stressed. | 0  (0) | 25  (3.5) | 212  (29.3) | 373  (51.5) | 114  (15.7) | 3.80  (.740) |
| #22 | I allowed the patient to take rest and calm himself/herself down. | 0  (0) | 15  (2.1) | 145  (20.0) | 416  (57.5) | 148  (20.4) | 3.96  (.698) |
| #24 | I shared nursing goals with other nursing staff members. | 1  (.1) | 20  (2.8) | 235  (32.5) | 372  (51.4) | 96  (13.3) | 3.75  (.719) |
| #21 | I assured the patient that hospitals are safe spaces. | 1  (.1) | 19  (2.6) | 201  (27.8) | 386  (53.3) | 117  (16.2) | 3.83  (.726) |
| #25 | I helped the patient feel more hopeful about community life. | 3  (.4) | 32  (4.4) | 301  (41.6) | 335  (46.3) | 53  (7.3) | 3.56  (.712) |
| **Excluded items** | | | | |  |  |  |
| #1 | I understood the whole context of the patient, considering the characteristics of schizophrenia. | 5  (.7) | 32  (4.4) | 272  (37.6) | 328  (45.3) | 87  (12.0) | 3.64  (.776) |
| #2 | I tried to be a sympathizer for the patient. | 3  (.4) | 25  (3.5) | 242  (33.4) | 379  (52.3) | 75  (10.4) | 3.69  (.719) |
| #3 | I knew that I could not understand everything about the patient on my own. | 7  (1.0) | 54  (7.5) | 245  (33.8) | 326  (45.0) | 92  (12.7) | 3.61  (.837) |
| #19 | I shared nursing goals with other nursing staff members. | 1  (.1) | 30  (4.1) | 220  (30.4) | 373  (51.5) | 100  (13.8) | 3.75  (.746) |
| #20 | I tried to relieve the suffering of the patient, considering that the patients were not good at getting along with people. | 2  (.3) | 42  (5.8) | 286  (39.5) | 301  (41.6) | 93  (12.8) | 3.61  (.793) |
| #27 | I encouraged the patient to reappraise his/her weaknesses as strengths. | 2  (.3) | 42  (5.8) | 258  (35.6) | 346  (47.8) | 76  (10.5) | 3.62  (.760) |
| #36 | I tried to notice changes in the patient’s attitudes toward medication. | 1  (.1) | 43  (5.9) | 246  (34.0) | 320  (44.2) | 114  (15.7) | 3.69  (.809) |

Abbreviations: IRERSS = In-hospital nursing care leading to reduction in early readmission among patients with schizophrenia scale, SD = standard deviation
