# Supplementary material for: Structure and predictors of in-hospital nursing care leading to reduction in early readmission among patients with schizophrenia in Japan: A cross-sectional study
Source: PLoS One. 2021 Apr 30;16(4):e0250771. doi: 10.1371/journal.pone.0250771 (PMC8087037; doi:10.1371/journal.pone.0250771)
Supplement: S1 Appendix — (DOCX) [file pone.0250771.s004.docx]

**Please recall a patient with schizophrenia who had previously been readmitted within 90 days of discharge but could live in a community for more than 90 days after receiving an in-hospital intervention. Circle the numbers (1-5) that apply to the nursing practice you performed for the patient.**

More than 90 days

In-hospital

Community

**In-hospital intervention**

Community

Less than 90days

**This nursing practices**

Strongly

agree

Strongly

disagree

1・・・・・2・・・・・・3・・・・・・4・・・・・・5

| 1. | I tried to understand the whole context of the patient, considering the characteristics of schizophrenia. | 1 | 2 | 3 | 4 | 5 |
| --- | --- | --- | --- | --- | --- | --- |
| 2. | I tried to be a sympathizer for the patient. | 1 | 2 | 3 | 4 | 5 |
| 3. | I knew that I could not understand everything about the patient on my own. | 1 | 2 | 3 | 4 | 5 |
| 4. | I often cooperated with other nursing staff members. | 1 | 2 | 3 | 4 | 5 |
| 5. | I shared important information about the patient with other nursing staff members. | 1 | 2 | 3 | 4 | 5 |
| 6. | As a nurse, I paid close attention to the things about which the patient was worried. | 1 | 2 | 3 | 4 | 5 |
| 7. | I tried to understand the challenges that the patient had been facing based on my observations of his/her daily life. | 1 | 2 | 3 | 4 | 5 |
| 8. | I tried to gain a more detailed understanding about why the patient was readmitted. | 1 | 2 | 3 | 4 | 5 |
| 9. | I tried to understand the patient’s capabilities and the challenges that he/she had been facing. | 1 | 2 | 3 | 4 | 5 |
| 10. | I envisioned nursing goals that aimed to enhance the post-discharge well-being of the patient. | 1 | 2 | 3 | 4 | 5 |
| 11. | I created nursing plans that incorporated the opinions of other nursing staff members. | 1 | 2 | 3 | 4 | 5 |
| 12. | I valued the happiness of the patient and his/her family. | 1 | 2 | 3 | 4 | 5 |
| 13. | I relieved the anxiety that the patient and his/her family experienced about community life. | 1 | 2 | 3 | 4 | 5 |
| 14. | I discussed with the patient and his/her family what he/her wanted to do in a community. | 1 | 2 | 3 | 4 | 5 |
| 15. | I discussed with the patient and his/her family how he/her can adapt to community life. | 1 | 2 | 3 | 4 | 5 |
| 16. | I helped the patient practice what he/she was not good at. | 1 | 2 | 3 | 4 | 5 |
| 17. | I helped the patient prepare for community life. | 1 | 2 | 3 | 4 | 5 |
| 18. | I reassured the patient’s family about the patient’s ability to adapt to community life after discharge. | 1 | 2 | 3 | 4 | 5 |
| 19. | I shared nursing goals with other nursing staff members. | 1 | 2 | 3 | 4 | 5 |
| 20. | I tried to relieve the suffering of the patient, considering that the patients were not good at getting along with people. | 1 | 2 | 3 | 4 | 5 |
| 21. | I assured the patient that hospitals are safe spaces. | 1 | 2 | 3 | 4 | 5 |
| 22. | I allowed the patient to take rest and calm himself/herself down. | 1 | 2 | 3 | 4 | 5 |
| 23. | I created spaces within which the patient did not feel stressed. | 1 | 2 | 3 | 4 | 5 |
| 24. | I valued the multifaceted opinions of other nursing staff. | 1 | 2 | 3 | 4 | 5 |
| 25. | I helped the patient feel more hopeful about community life. | 1 | 2 | 3 | 4 | 5 |
| 26. | I helped the patient accept his/her disability. | 1 | 2 | 3 | 4 | 5 |
| 27. | I encouraged the patient to reappraise his/her weaknesses as strengths. | 1 | 2 | 3 | 4 | 5 |
| 28. | I helped the patient improve his/her lifestyle. | 1 | 2 | 3 | 4 | 5 |
| 29. | I helped the patient reconsider his/her thoughts so that he/she could realize that his/her delusions were thoughts that are inconsistent with reality. | 1 | 2 | 3 | 4 | 5 |
| 30. | I talked with the patient about how to deal with delusions so that he/she could take responsibility for behaviors. | 1 | 2 | 3 | 4 | 5 |
| 31. | I evaluated my nursing care from changes in the patient’s behaviors. | 1 | 2 | 3 | 4 | 5 |
| 32. | I believed in the patient and encourage him/her to change behaviors. | 1 | 2 | 3 | 4 | 5 |
| 33. | I observed how the patient deals with delusions. | 1 | 2 | 3 | 4 | 5 |
| 34. | I helped the patient understand the need for medication by providing factual information. | 1 | 2 | 3 | 4 | 5 |
| 35. | I tried to notice changes in the patient’s attitudes toward medication. | 1 | 2 | 3 | 4 | 5 |
| 36. | I cherished the way the patient wanted to live. | 1 | 2 | 3 | 4 | 5 |
| 37. | I was in touch with the patient and his/her caregivers, for a while, even after discharge. | 1 | 2 | 3 | 4 | 5 |
| 38. | I put the patient in touch with community nurses to ensure the continuity of care. | 1 | 2 | 3 | 4 | 5 |
| 39. | I discussed with the patient the good things that could happen to him/her after discharge with him/her. | 1 | 2 | 3 | 4 | 5 |
| 40. | I participated in care conferences that involved community care providers. | 1 | 2 | 3 | 4 | 5 |
| 41. | I assessed the patient’s self-care abilities by comparing his/her behaviors at the time of admission and discharge. | 1 | 2 | 3 | 4 | 5 |
| 42. | I discussed with the patient the services that he/she wanted to use in his/her community. | 1 | 2 | 3 | 4 | 5 |
| 43. | I informed the patient and his/her family about the support system that would be available to the patient after discharge. | 1 | 2 | 3 | 4 | 5 |
