# Supplementary material for: Structure and predictors of in-hospital nursing care leading to reduction in early readmission among patients with schizophrenia in Japan: A cross-sectional study
Source: PLoS One. 2021 Apr 30;16(4):e0250771. doi: 10.1371/journal.pone.0250771 (PMC8087037; doi:10.1371/journal.pone.0250771)
Supplement: S2 Appendix — (DOCX) [file pone.0250771.s005.docx]

**過去に関わった統合失調症患者のうち，90日以内に再入院し，入院治療の後に90日を超えて地域生活を送った患者を思い浮かべてください。その患者にあなたが行った看護実践について，該当する番号（1～5）の1つに〇をつけてください。**

90日を

超える

入院

地域

**再入院**

地域

90日

未満

**ここでの**

**看護実践**

かなり

当てはまる

全く当て

はまらない

1・・・・・2・・・・・・3・・・・・・4・・・・・・5

| 1. | 統合失調症の特性を踏まえた患者の人となりをつかみ，全体像を思い描く | 1 | 2 | 3 | 4 | 5 |
| --- | --- | --- | --- | --- | --- | --- |
| 2. | 統合失調症患者の思いを慮り，患者の理解者となる | 1 | 2 | 3 | 4 | 5 |
| 3. | 看護師の自分一人では統合失調症患者の課題をつかむことができない状況を捉えている | 1 | 2 | 3 | 4 | 5 |
| 4. | 退院支援に向け，日頃からスタッフ間の助け合いを意識している | 1 | 2 | 3 | 4 | 5 |
| 5. | 統合失調症患者が話しやすい看護師を通して，スタッフ間で情報を共有する | 1 | 2 | 3 | 4 | 5 |
| 6. | 日常的に関わる看護師として，統合失調症患者が困ることを普段から把握しておく | 1 | 2 | 3 | 4 | 5 |
| 7. | 印象的な出来事や気にかかる生活状況を通して明らかになる統合失調症患者の課題に気づく | 1 | 2 | 3 | 4 | 5 |
| 8. | 以前の入院の時よりも詳しく，統合失調症患者の再入院に至るプロセスに関する情報をつかむ | 1 | 2 | 3 | 4 | 5 |
| 9. | 統合失調症患者の残存している能力をつかみ，再入院の原因と患者の課題をつきとめる | 1 | 2 | 3 | 4 | 5 |
| 10. | 統合失調症患者の退院後の生活をイメージし，退院後の生活に向けた目標を想定する | 1 | 2 | 3 | 4 | 5 |
| 11. | 他のスタッフの多角的な意見を集約し，看護の方向性を明確にする | 1 | 2 | 3 | 4 | 5 |
| 12. | 統合失調症患者と家族の関係性から，双方にとっての幸せを考える | 1 | 2 | 3 | 4 | 5 |
| 13. | 家族と統合失調症患者の歴史に思いを巡らし，家族の退院への抵抗感を和らげる | 1 | 2 | 3 | 4 | 5 |
| 14. | 家族と信頼関係を結び，患者と家族と看護師の三者で退院後の生活の目標を共有する | 1 | 2 | 3 | 4 | 5 |
| 15. | 退院後，社会資源を利用しながら継続してゆける生活について，家族と相談する | 1 | 2 | 3 | 4 | 5 |
| 16. | 退院後の統合失調症患者の新しい居場所を想定し，病院内で行えることを緩やかに準備する | 1 | 2 | 3 | 4 | 5 |
| 17. | 統合失調症患者や家族に対し，単身生活や施設入所という環境変化への抵抗感を和らげる | 1 | 2 | 3 | 4 | 5 |
| 18. | 退院に向けて，統合失調症患者や家族の緊張感に対して，心の準備をすすめていく | 1 | 2 | 3 | 4 | 5 |
| 19. | 統合失調症患者に振り返りを促しながら，目標を共有する | 1 | 2 | 3 | 4 | 5 |
| 20. | 統合失調症の特性であるラポール形成能力の障害を踏まえ，患者の対人関係における苦悩を軽減したい  という願いをもって関わる | 1 | 2 | 3 | 4 | 5 |
| 21. | 統合失調症患者の他者への緊張に対して，安全感を保証し，患者との信頼関係を形成する | 1 | 2 | 3 | 4 | 5 |
| 22. | 統合失調症患者の不安定な状態に対して，休養を促し症状の鎮静化をはかる | 1 | 2 | 3 | 4 | 5 |
| 23. | 統合失調症患者に対して，ストレスを与えないよう配慮する | 1 | 2 | 3 | 4 | 5 |
| 24. | 他のスタッフの多角的な意見を網羅した統一的な関わりをする | 1 | 2 | 3 | 4 | 5 |
| 25. | 統合失調症患者の望みを具体的な地域生活につなげて，ストレスがかからないようにする | 1 | 2 | 3 | 4 | 5 |
| 26. | 統合失調症を病気として受け入れ生活する方法を探す | 1 | 2 | 3 | 4 | 5 |
| 27. | 統合失調症患者の弱さを強みと捉え,患者が退院後も自信をもって継続できる生活習慣を身につけられるように支える | 1 | 2 | 3 | 4 | 5 |
| 28. | 統合失調症患者の生活習慣の改善を図り，患者自身で生活習慣を維持できるか，評価する | 1 | 2 | 3 | 4 | 5 |
| 29. | 統合失調症患者に振り返りを促すことで，誤った外界に対する認知に気づきを与える | 1 | 2 | 3 | 4 | 5 |
| 30. | 統合失調症患者が気づいた認知の誤りへの対処を，より具体化し，自分の行動に責任感をもつように援助する | 1 | 2 | 3 | 4 | 5 |
| 31. | 統合失調症患者のとる対処行動が変化し始めた兆しから，看護師の行った援助を評価する | 1 | 2 | 3 | 4 | 5 |
| 32. | 陽性症状の悪化に気をつけながら統合失調症患者の変容を強化し，患者自身の問題解決力を信じる | 1 | 2 | 3 | 4 | 5 |
| 33. | 統合失調症患者が認知の誤りへの対処を身につけたことを確認する | 1 | 2 | 3 | 4 | 5 |
| 34. | 統合失調症患者に振り返りを促して，自己認知の違和感を具体化し，薬の必要性を自覚できる気づきを与える | 1 | 2 | 3 | 4 | 5 |
| 35. | 統合失調症患者の陽性症状悪化時の自己の認識や薬に対する認識の変化を評価する | 1 | 2 | 3 | 4 | 5 |
| 36. | 入院中の看護は患者の人生にとってプロセスの一部と捉え，患者の望む生き方を尊重する | 1 | 2 | 3 | 4 | 5 |
| 37. | 退院後，規則正しい生活スタイルを継続していけるよう，統合失調症患者と周りの人を継続的にフォローする | 1 | 2 | 3 | 4 | 5 |
| 38. | 地域で関わる看護師へ，患者の退院後に，入院中の支援を続けられるように橋渡しをする | 1 | 2 | 3 | 4 | 5 |
| 39. | 統合失調症患者に対して，退院すると，どのようなメリットがあるかを伝える | 1 | 2 | 3 | 4 | 5 |
| 40. | 退院前に訪問看護やホームヘルパーが参加する多職種でのケア会議に参加する | 1 | 2 | 3 | 4 | 5 |
| 41. | 入院中と退院後の統合失調症患者の状態を比較し，セルフケア能力の差異を査定する | 1 | 2 | 3 | 4 | 5 |
| 42. | 統合失調症患者とともに，退院後に患者が利用する社会資源を一緒に見たり，感想を話し合ったりする | 1 | 2 | 3 | 4 | 5 |
| 43. | 退院後の支援体制を医療チーム間および統合失調症患者とその家族で確認をする | 1 | 2 | 3 | 4 | 5 |
