# Supplementary material for: Structure and predictors of in-hospital nursing care leading to reduction in early readmission among patients with schizophrenia in Japan: A cross-sectional study
Source: PLoS One. 2021 Apr 30;16(4):e0250771. doi: 10.1371/journal.pone.0250771 (PMC8087037; doi:10.1371/journal.pone.0250771)
Supplement: S3 Appendix — (DOCX) [file pone.0250771.s006.docx]

**Please circle the items that apply to the hospital / ward where you are working.**

| 1. | Chose the establishment of the hospital where you are working | 1. Public |
| --- | --- | --- |
|  |  | 2. No-profit corporation |
|  |  | 3. Private |
| 2. | Does your ward adopt primary nursing? | 1. Yes |
|  |  | 2. No |
| 3. | Does your ward usually hold pre-discharge conferences? *(Choose the one that best applies.)* | 1. Yes |
|  |  | 1. No |
| 4. | **Answer if you selected “1. Yes” for Question 3.**  [Participation of families in pre-discharge conferences]  Do patients’ families usually participate in predischarge conferences?  *(Choose the most common case.)* | 1. No |
|  |  | 1. Yes |
| 5. | **Answer if you selected “1. Yes” for Question 3.**  [Participation of multidisciplinary teams in pre-discharge conferences]  What members consist of predischarge conferences? *(Choose the most common case.)* | 1. Consist only of hospital staff |
|  |  | 2. Consist of both community and hospital staff |

**Please circle the items that apply to you. If applicable, write in parentheses.**

| 1. | Choose your gender. | 1. Male | | 2. Female | |
| --- | --- | --- | --- | --- | --- |
| 2. | Do you have a certified nurse qualification? | 1. No | 2. Yes  →　Field（ 　　　　　　） | | |
| 3. | Do you have a certified nurse specialist qualification? | 1. No | 2. Yes  →　Field（　 　　　　） | | |
| 4. | Do you have practical experience as a home visiting nurse? | 1. Yes | | | 1. No |
| 5. | Do you have practical experience in providing psychiatric outpatient care? | 1. Yes | | | 1. No |
| 6. | How long have you worked in a psychiatric ward? | （　　　　　　）years（　　　　　　　）months | | | |
| 7. | Do you have practical experience in somatic care wards? | 1. Yes | | | 1. No |
| 8. | Choose your educational level. | 1. Diploma | | | |
|  |  | 2. Bachelor | | | |
|  |  | 3. Master | | | |
|  |  | 4. Other （　　　　　　　　　　　　　　　　） | | | |
