# Supplementary material for: Structure and predictors of in-hospital nursing care leading to reduction in early readmission among patients with schizophrenia in Japan: A cross-sectional study
Source: PLoS One. 2021 Apr 30;16(4):e0250771. doi: 10.1371/journal.pone.0250771 (PMC8087037; doi:10.1371/journal.pone.0250771)
Supplement: S4 Appendix — (DOCX) [file pone.0250771.s007.docx]

**あなたの勤務する病院/病棟について，該当するものに○をつけて下さい。**

| 1. | あなたの勤務する病院の設置主体を選んでください。 | 1. 公的病院・公立病院 |
| --- | --- | --- |
|  |  | 2. 公益法人 |
|  |  | 3. 医療法人 |
| 2. | あなたの勤務する病棟では、プライマリーナーシングを採用していますか？ | 1. はい |
|  |  | 2. いいえ |
| 3. | 病棟の退院前カンファレンスの実施状況を選んでください。　　　　　（**最も当てはまるもの1つに**） | 1. 行っている |
|  |  | 1. 行っていない |
| 4. | **3.**の質問で「1.行っている」と回答した方へ  【患者の家族のカンファレンス参加に関して】  退院前カンファレンスについて、病棟で最も多い事例を選んでください。 （**最も当てはまるもの1つに**） | 1. 家族には参加してもらっていない |
|  |  | 1. 家族には参加してもらう |
| 5. | **3.**の質問で「1.行っている」と回答した方へ  【医療・福祉職のカンファレンス参加に関して】  退院前カンファレンスについて、病棟で最も多い事例を選んでください。 （**最も当てはまるもの1つに**） | 1. 病院の多職種が参加するカンファレンスを行う |
|  |  | 2. 患者の退院後に地域で関わる医療職・福祉職を含めて  カンファレンスを行う |

**あなたのことについて，当てはまる番号に○をつけるか，カッコ内に記入をして下さい。**

| 1. | 性別を選んでください。 | 1. 男性 | | 2. 女性 | |
| --- | --- | --- | --- | --- | --- |
| 2. | 認定看護師の資格を持っていますか？ | 1. いいえ | 2. はい  →　分野（ 　　　　　　） | | |
| 3. | 専門看護師の資格を持っていますか？ | 1. いいえ | 2. はい  →　分野（　 　　　　） | | |
| 4. | 精神科訪問看護での実務経験はありますか？ | 1. あり | | | 1. なし |
| 5. | 精神科外来での実務経験はありますか？ | 1. あり | | | 1. なし |
| 6. | 精神科病棟での勤務経験は何年何カ月ですか？ | （　　　　　　）年（　　　　　　　）カ月 | | | |
| 7. | 精神科以外の診療科での勤務経験はありますか？ | 1. あり | | | 1. なし |
| 8. | 最終学歴を選んでください。 | 1. 高等学校・専門学校 | | | |
|  |  | 2. 大学 | | | |
|  |  | 3. 大学院（修士課程） | | | |
|  |  | 4. その他　（　　　　　　　　　　　　　　　　） | | | |
