# Supplementary material for: Structure and predictors of in-hospital nursing care leading to reduction in early readmission among patients with schizophrenia in Japan: A cross-sectional study
Source: PLoS One. 2021 Apr 30;16(4):e0250771. doi: 10.1371/journal.pone.0250771 (PMC8087037; doi:10.1371/journal.pone.0250771)
Supplement: Attachment — Submitted filename: PONE-D-20-29533_reviewer.pdf [file pone.0250771.s008.pdf]

PLOS ONE

Structure and predictors of in-hospital nursing care to prevent early readmission in patients with schizophrenia in Japan: A cross-sectional study

--Manuscript Draft--

**Manuscript Number:**	PONE-D-20-29533
**Article Type:**	Research Article
**Full Title:**	Structure and predictors of in-hospital nursing care to prevent early readmission in patients with schizophrenia in Japan: A cross-sectional study
**Short Title:**	In-hospital nursing care to prevent early readmission in patients with schizophrenia
**Corresponding Author:**	Shigeyoshi Maki<br>Sugiyama Jogakuen University: Sugiyama Jogakuen Daigaku<br>Nagoya, Aichi JAPAN
**Keywords:**	readmission; schizophrenia; psychiatric nursing; continuity of care; exploratory factor analysis; regression analysis
**Abstract:**	Schizophrenia is a disorder characterized by psychotic relapses. Some patients with schizophrenia are re-admitted to hospitals when their condition worsens so that they cannot live in communities. The purpose of this study was to investigate the structure and predictors of in-hospital nursing care to prevent early readmission in patients with schizophrenia.<br>A new questionnaire was developed to assess the extent to which respondents delivered in-hospital nursing care to prevent early readmission in patients with schizophrenia. This study adopted a cross-sectional research design. The survey was conducted with the new questionnaires. The participants were registered nurses working in psychiatric wards. Item analyses and exploratory factor analyses were performed using the new questionnaires to investigate the structure of in-hospital nursing care to prevent early readmission in patients with schizophrenia. Stepwise regression analyses were conducted to examine the factors predicting in-hospital nursing care to prevent early readmission.<br>Data were collected from 724 registered nurses in Japan. In-hospital nursing care to prevent early readmission was found to consist of five factors: promoting cognitive functioning and self-care, identifying reasons for readmission, establishing cooperative systems within the community, sharing goals about community life, and creating restful spaces. In-hospital nursing care to prevent early readmission was predicted by the following variables: the score on the nursing excellence scale in clinical practice, the score on therapeutic hold, and the participation of community care providers in pre-discharge conferences.<br>Japanese psychiatric nurses provide nursing care based on these five factors to prevent early readmission in patients with schizophrenia. Such nursing care would be facilitated by not only nurses' excellence but also nurses' environmental factors, especially the therapeutic climate of the ward and the participation of community care providers in pre-discharge conferences.
**Order of Authors:**	Shigeyoshi Maki
	Kuniyoshi Nagai
	Shoko Ando
	Koji Tamakoshi
**Additional Information:**	
**Question**	**Response**
**Financial Disclosure**<br><br>Enter a financial disclosure statement that describes the sources of funding for the work included in this submission. Review	SM was funded by Japan Society for the Promotion of Science KAKENHI Grant# JP17K17523.<br>URL: https://kaken.nii.ac.jp/grant/KAKENHI-PROJECT-17K17523/<br>The funders had no role in study design, data collection and analysis, decision to publish, or preparation of the manuscript.

Powered by Editorial Manager® and ProduXion Manager® from Aries Systems Corporation

the submission guidelines for detailed requirements. View published research articles from *PLOS ONE* for specific examples.<br><br>This statement is required for submission and **will appear in the published article** if the submission is accepted. Please make sure it is accurate.<br><br>**Unfunded studies**<br>Enter: *The author(s) received no specific funding for this work.*<br><br>**Funded studies**<br>Enter a statement with the following details:<br>• Initials of the authors who received each award<br>• Grant numbers awarded to each author<br>• The full name of each funder<br>• URL of each funder website<br>• Did the sponsors or funders play any role in the study design, data collection and analysis, decision to publish, or preparation of the manuscript?<br>• **NO** - Include this sentence at the end of your statement: *The funders had no role in study design, data collection and analysis, decision to publish, or preparation of the manuscript.*<br>• **YES** - Specify the role(s) played.<br><br>* typeset	
**Competing Interests**<br><br>Use the instructions below to enter a competing interest statement for this submission. On behalf of all authors, disclose any competing interests that could be perceived to bias this work—acknowledging all financial support and any other relevant financial or non-financial competing interests.<br><br>This statement **will appear in the published article** if the submission is accepted. Please make sure it is accurate. View published research articles from *PLOS ONE* for specific examples.	The authors have declared that no competing interests exist.

Powered by Editorial Manager® and ProduXion Manager® from Aries Systems Corporation

**NO authors have competing interests**<br><br>Enter: *The authors have declared that no competing interests exist.*<br><br>**Authors with competing interests**<br><br>Enter competing interest details beginning with this statement:<br><br>*I have read the journal's policy and the authors of this manuscript have the following competing interests: [insert competing interests here]*<br><br>* typeset	
**Ethics Statement**<br><br>Enter an ethics statement for this submission. This statement is required if the study involved:<br><br>• Human participants<br>• Human specimens or tissue<br>• Vertebrate animals or cephalopods<br>• Vertebrate embryos or tissues<br>• Field research<br><br>Write "N/A" if the submission does not require an ethics statement.<br><br>General guidance is provided below. Consult the submission guidelines for detailed instructions. **Make sure that all information entered here is included in the Methods section of the manuscript.**	Ethical approval to conduct this study was granted by the ethics committee of the Graduate School of Medicine, Nagoya University, Japan (No: 17-155). The participants were informed about the aims of the study and the benefits and risks of participation through printed forms. The participants provided written informed consent. They responded to each questionnaire anonymously, enclosed the completed questionnaires in sealed envelopes, and returned them to the researchers. Hospital directors and nurse managers did not participate in the data collection process. If participants had any questions about the study, they were able to call or email the principal investigator, whose phone number and address were listed on the documents for research cooperation. Permissions to use the EssenCES-JPN and the NES were obtained from the copyright holders.

Powered by Editorial Manager® and ProduXion Manager® from Aries Systems Corporation

**Format for specific study types**<br><br>**Human Subject Research (involving human participants and/or tissue)**<br>• Give the name of the institutional review board or ethics committee that approved the study<br>• Include the approval number and/or a statement indicating approval of this research<br>• Indicate the form of consent obtained (written/oral) or the reason that consent was not obtained (e.g. the data were analyzed anonymously)<br><br>**Animal Research (involving vertebrate animals, embryos or tissues)**<br>• Provide the name of the Institutional Animal Care and Use Committee (IACUC) or other relevant ethics board that reviewed the study protocol, and indicate whether they approved this research or granted a formal waiver of ethical approval<br>• Include an approval number if one was obtained<br>• If the study involved *non-human primates*, add *additional details* about animal welfare and steps taken to ameliorate suffering<br>• If anesthesia, euthanasia, or any kind of animal sacrifice is part of the study, include briefly which substances and/or methods were applied<br><br>**Field Research**<br><br>Include the following details if this study involves the collection of plant, animal, or other materials from a natural setting:<br>• Field permit number<br>• Name of the institution or relevant body that granted permission	
**Data Availability**<br><br>Authors are required to make all data underlying the findings described fully available, without restriction, and from the time of publication. PLOS allows rare exceptions to address legal and ethical concerns. See the PLOS Data Policy and FAQ for detailed information.	Yes - all data are fully available without restriction

A Data Availability Statement describing where the data can be found is required at submission. Your answers to this question constitute the Data Availability Statement and **will be published in the article**, if accepted.<br><br>**Important:** Stating 'data available on request from the author' is not sufficient. If your data are only available upon request, select 'No' for the first question and explain your exceptional situation in the text box.<br><br>Do the authors confirm that all data underlying the findings described in their manuscript are fully available without restriction?	
**Describe where the data may be found in full sentences. If you are copying our sample text, replace any instances of XXX with the appropriate details.**<br><br>• If the data are **held or will be held in a public repository**, include URLs, accession numbers or DOIs. If this information will only be available after acceptance, indicate this by ticking the box below. For example: *All XXX files are available from the XXX database (accession number(s) XXX, XXX.).*<br>• If the data are all contained **within the manuscript and/or Supporting Information files**, enter the following: *All relevant data are within the manuscript and its Supporting Information files.*<br>• If neither of these applies but you are able to provide **details of access elsewhere**, with or without limitations, please do so. For example:<br><br>*Data cannot be shared publicly because of [XXX]. Data are available from the XXX Institutional Data Access / Ethics Committee (contact via XXX) for researchers who meet the criteria for access to confidential data.*<br><br>*The data underlying the results presented in the study are available from (include the name of the third party*	All relevant data are within the manuscript and its Supporting Information files.

and contact information or URL).<br>• This text is appropriate if the data are owned by a third party and authors do not have permission to share the data.<br><br>* typeset	
Additional data availability information:	

Powered by Editorial Manager® and ProduXion Manager® from Aries Systems Corporation

Manuscript Click here to access/download;Manuscript;Manuscript.docx

Structure and predictors of in-hospital nursing care to prevent early readmission in patients with schizophrenia in Japan: A cross-sectional study

Shigeyoshi Maki[1*¶], Kuniyoshi Nagai[2¶], Shoko Ando[3¶], and Koji Tamakoshi[3¶]

[1] Department of Nursing, School of Nursing, Sugiyama Jogakuen University, Nagoya, Aichi, Japan

[2] Department of Nursing, School of Nursing, Nagoya University of Arts and Sciences, Nagoya, Aichi, Japan

[3] Department of Nursing, Nagoya University Graduate School of Medicine (Health Sciences), Nagoya, Aichi, Japan

*Corresponding author

E-mail: s-maki@sugiyama-u.ac.jp (SM)

[¶] These authors contributed equally to this work.

// Abstract

Schizophrenia is a disorder characterized by psychotic relapses. Some patients with schizophrenia are re-admitted to hospitals when their condition worsens so that they cannot live in communities. The purpose of this study was to investigate the structure and predictors of in-hospital nursing care to prevent early readmission in patients with schizophrenia.

A new questionnaire was developed to assess the extent to which respondents delivered in-hospital nursing care to prevent early readmission in patients with schizophrenia. This study adopted a cross-sectional research design. The survey was conducted with the new questionnaires. The participants were registered nurses working in psychiatric wards. Item analyses and exploratory factor analyses were performed using the new questionnaires to investigate the structure of in-hospital nursing care to prevent early readmission in patients with schizophrenia. Stepwise regression analyses were conducted to examine the factors predicting in-hospital nursing care to prevent early readmission.

Data were collected from 724 registered nurses in Japan. In-hospital nursing care to prevent early readmission was found to consist of five factors: promoting cognitive functioning and self-care, identifying reasons for readmission, establishing cooperative systems within the community, sharing goals about community life, and creating restful spaces. In-hospital nursing care to prevent early readmission was predicted by the following variables: the score on the nursing excellence scale in clinical practice, the score on therapeutic hold, and the participation of community care providers in pre-discharge conferences.

Japanese psychiatric nurses provide nursing care based on these five factors to prevent early readmission in patients with schizophrenia. Such nursing care would be facilitated by not only nurses' excellence but also nurses' environmental factors, especially the therapeutic climate of the ward and the participation of community care providers in pre-discharge conferences.

Introduction

Schizophrenia is a disorder characterized by psychotic relapses [1]. Many people with schizophrenia suffer recurrent symptoms of psychosis (i.e., hallucinations, delusions, and disorganized speech) and chronic cognitive deficits (i.e., impaired executive function, memory, and speed of mental processing) [2]. Various indicators are used for relapse in schizophrenia. Readmission rates are widely used as an indicator for relapse among schizophrenia patients [3, 4]. Some patients with schizophrenia are readmitted to hospitals when their condition worsens so that they cannot live in communities [2]. Readmissions lead to rising costs of mental health care [3-5]. Furthermore, readmissions put a strain on patients, affect their prognosis, and lead to a breakdown in their family function [3, 6].

Several studies examined the factors that contribute to readmission. Schizophrenia is one of the diagnoses of mental disorders associated with psychiatric readmission [7-9]. Factors associated with readmission can be classified based on three perspectives: patients' demographic characteristics, patients' clinical characteristics, and patients' social characteristics. Regarding patients' demographic characteristics, younger age [10, 11] and non-marital status [11] are associated with increased risk for readmission. Regarding patients' clinical characteristics, complications [6, 12], a history of previous hospitalizations [6, 11, 12], involuntary admission days [11, 12], the use of multiple health services [6], total admission days [6, 11], unplanned discharges [12], the proportion of experienced psychiatrists at a hospital [13], and medication non-adherence [14-16] are associated with increased risk for readmission. Regarding patients' social characteristics, maladaptive functioning of family systems [17] is associated with increased risk for readmission. Furthermore, a length of stay in a psychiatric ward of more than 28 days plays a protective role in preventing readmission [4].

In particular, readmissions within 90 days of discharge are usually defined as early re-admissions [10]. Between 15%–30% of patients with psychiatric disorders experience early

readmission [4, 11, 18, 19]. Early readmissions reflect the degree of continuity between hospital care and community care [10]. Patients take an average of 90 days to establish therapeutic relationships with community care providers after their discharge [20]. Many patients encounter critical situations within 90 days of discharge [21, 22]. The prevention of early readmissions could lead to successful transitions of patients from hospital care to community care. However, the structure and predictors of in-hospital nursing care to prevent early readmission in patients with schizophrenia are unclear.

Most psychiatric care in Japan is still provided in inpatient settings [23], despite Japan's efforts to facilitate the transition from inpatient care to community care [24]. The number of psychiatric beds in Japan is four times higher than the Organization for Economic Cooperation and Development (OECD) average (267 beds vs. 66 beds per 100,000 population) [25]. The average length of stay in a psychiatric hospital in Japan is approximately 300 days, much longer than in other OECD countries [26, 27]. Community mental health services in Japan are not sufficient [27]. Approximately 25% of inpatients experience readmissions within 90 days of discharge (i.e., early readmissions) [28]. In Japan, delivering community mental health services for patients in communities are not enough to prevent early readmission in patients [4]. Japanese hospital nurses need to be more proactive in providing nursing care to prevent early readmission in patients, so that the patients can spend their lives more comfortably after discharge. However, the predictors of in-hospital nursing care to prevent early readmissions are unknown.

In-hospital nursing care to prevent early readmission may be predicted by nurses' environmental factors as well as their individual factors. The risk of early readmission could be predicted by not only community follow-ups but also by length of stay in hospital [4]. Nursing performance is influenced by nurses' environmental factors as well as personal factors [29]. A positive social climate in hospitals is associated with higher patient treatment

motivation, treatment engagement, and patient-nurse therapeutic alliance [30]. A significant relationship between social climate and staff performance and morale has been reported [31]. We hypothesized that in-hospital nursing care to prevent early readmission could also be predicted by the social climate in psychiatric wards as well as by nurses' individual factors.

The purpose of this study was: (a) to investigate the structure that underlies in-hospital nursing care to prevent early readmission in patients with schizophrenia; and (b) to examine the factors predicting in-hospital nursing care to prevent early readmission, by focusing on both the social climate in psychiatric wards and nurses' individual factors.

Methods

Design

This study adopted a cross-sectional research design and utilized self-administered questionnaires, which participants completed anonymously.

Settings and participants

This survey was conducted at hospitals where psychiatric beds accounted for more than 60% of all beds. The hospitals were selected in three steps. First, the hospitals were extracted from the Japan Medical Analysis Platform of the Japan Medical Association (http://jmap.jp/). Of the 1,271 hospitals extracted, 59 were public hospitals, 69 were non-profit corporation hospitals, and 1,143 were private hospitals. Second, the 128 public hospitals and hospitals owned by non-profit corporations were all included in the study, as well as a randomly selected sample of 128 private hospitals, which totaled 256 hospitals. Third, we then mailed request documents for research cooperation to the directors of the 256 hospitals. Written consents for research cooperation were obtained from 40 hospitals (i.e., 19 public hospitals, six hospitals owned by non-profit corporations, and 15 private hospitals).

Participants were 1,995 registered nurses (RNs) working in the 40 hospitals. Self-administered questionnaires were mailed to all the RNs. The RNs, who gave written consent for research cooperation, returned the completed questionnaires to the researchers by mail. The inclusion criteria were: 1) full-time RN, and 2) working in a psychiatric ward. The exclusion criteria were: 1) nurse managers, 2) RNs working in outpatient wards, and 3) part-time nurses. Data were collected between February and March 2018.

Sample size estimations were based on the recommendations of Pett, Lackey & Sullivan [32]. They recommended that the minimum number of subjects for an exploratory factor analysis (EFA) was 10 to 15 per initial item. In this study, 430–645 subjects were required. Considering a valid response rate of approximately 30%, 1,433–2,150 questionnaires had to be distributed.

Measurement

Demographic data

The following demographic characteristics of the participants were examined: gender, years of psychiatric experience, advanced practice registered nurse (APRN) status (certified nurse or certified nurse specialist), experience as a psychiatric home-visiting nurse, experience in providing psychiatric outpatient care, experience in somatic care wards, and educational level. Further, the following characteristics of the hospitals or wards in which they were working were asked: hospital establishment, adoption of primary nursing, whether or not pre-discharge conferences were usually held, the participation of patients' families in the pre-discharge conferences, and the participation of multidisciplinary teams in the pre-discharge conferences. Only participants who answered that "pre-discharge conferences were usually held" responded to the question if the patients' families attended the pre-discharge conferences. Similarly, only participants who explained that "pre-discharge conferences were usually held"

responded to the question of whether the conference member consisted only of hospital staff or consisted of both community and hospital staff.

The in-hospital nursing care to prevent early readmission in patients with schizophrenia scale (IPERSS)

A 43-item IPERSS was developed in this study. In the IPERSS, respondents were required to recall a patient with schizophrenia who had previously been readmitted within 90 days of discharge, but who could live in a community for more than 90 days after receiving an in-hospital intervention. The respondents answered to what extent they provided the nursing care indicated in the questionnaire during the patient's in-hospital intervention (see S1 Figure.). It was easy for the respondents to recall patients who had experienced readmissions within 90 days of discharge, namely, early readmissions, because the Japanese public health insurance program defines hospitalization within 90 days of the discharge as a readmission and after more than 90 days after discharge as a new hospitalization [33]. This nursing care would be effective in preventing early readmissions if the patient was not re-hospitalized early after receiving the nursing care.

Responses to each item were recorded on a 5-point Likert scale that ranged from 1 (strongly disagree) to 5 (strongly agree). The items of the IPERSS were developed based on Maki et al.'s findings [34]. In their qualitative study, they examined in-hospital nursing care to prevent early readmission in patients by conducting interviews with 17 proficient psychiatric nurses. They revealed 38 concepts of nursing practice to prevent early readmission in patients with schizophrenia. Based on the 38 concepts, we created 38 items of in-hospital nursing care to prevent early readmission in patients with schizophrenia. Furthermore, five of the items (Items 39, 40, 41, 42, and 43) were added based on the feedback provided by the five experts (i.e., one researcher in the field of mental health nursing, one psychiatrist, and two experienced nursing researchers). To make the expression of the items clearer and more concise, they were

revised based on the opinions of a five-person panel (i.e., two graduate students, one university faculty member, and two psychiatric nurses).

The Japanese version of the Essen climate evaluation schema (EssnCES-JPN)

EssenCES is a 17-item questionnaire that measures three aspects of the social climate in psychiatric wards (PC: Patients' cohesion and mutual support, ES: Experienced safety, and TH: Therapeutic hold). Each subscale included five items, and two items were not included in any of the subscales. Each item used a 5-point Likert scale from 0 (not at all) to 4 (very much). Higher scores indicated that respondents perceived the hospital ward climate as more positive. "Patients' cohesion and mutual support" reflected an essential quality of therapeutic communities and effectively working treatment groups [35]. "Experienced safety" referred to the level of perceived tension and threat of aggression or violence [36]. "Therapeutic hold" indicated the extent to which the unit was perceived as supportive of patients' therapeutic needs [37]. The questionnaire was originally developed to assess the social and therapeutic atmosphere of forensic psychiatric wards [38]. This questionnaire's transferability to general psychiatric settings was confirmed [36]. The Japanese version of this scale had good internal consistency and construct validity [39, 40].

Nursing performance was influenced by not only nurses' individual factors but also environmental factors [29, 41]. This study investigated the relevance between in-hospital nursing care to prevent early readmissions and the environmental factors, using EssenCES-JPN.

Nursing excellence scale in clinical practice (NES)

The NES was developed in Japan [42]. Responses to each item were recorded on a 5-point Likert scale that ranged from 1 (not at all) to 5 (strongly agree). Higher scores were indicative of self-reported nursing excellence in clinical practice. The NES consists of 35 items and seven subscales, namely: (1) collecting and using client's information continuously and

efficiently, (2) performing with appropriate knowledge/skills in clinical settings, (3) developing relationships with clients/families through communication, (4) overcoming difficult conditions of clients and/or in the work environment, (5) identifying potential problems for clients and solving them creatively, (6) protecting the personality and human dignity of clients, and (7) being aware of fulfilling a number of roles as a medical and nursing team member and being able to perform them. The NES had good reliability and validity [42, 43]. The NES had similarities with the IPERSS in that nurses could easily assess their own nursing practice. The NES, which measures nurses' individual nursing practice, was used as one of the predictors of the IPERSS.

Data Analysis

All statistical analyses were conducted using IBM SPSS Statistics version 25. Questionnaires that contained missing responses were excluded. Descriptive analyses were performed to examine the demographic characteristics of the participants and the features of the hospitals/wards in which they worked. The normality of data was evaluated by means of a visual inspection of histograms and QQ-plots. All statistical tests were two-tailed and $p < .05$ was considered significant.

Latent structure of the IPERSS

First, item analyses were performed on the items of the IPERSS. Ceiling and floor effects were examined for each item of the IPERSS. Inter-item and item-total correlations were computed. In the inter-item correlation analyses, items with a correlation < .30 with all items, or items with a correlation > .90 with any item were removed to avoid the risk of multicollinearity [44]. In the item-total correlation analyses, an item was excluded if the correlation between the item score and total score without the respective item was <.30.

Second, an EFA was conducted using maximum likelihood extraction and promax rotation. The Kaiser-Guttman criterion (eigenvalues > 1) was used to determine the number of

factors that should be retained. Items with loadings that were lower than (i.e., < .40) onto all factors were excluded. Additionally, items that were strongly loaded (i.e., > .40) onto two or more factors were also excluded. The analysis was repeated until all the items were strongly loaded onto only a single factor. The adequacy of the EFA was evaluated by the Kaiser-Meyer-Olkin (KMO) statistic and Bartlett's test of sphericity. The internal consistency of the scale was examined by computing Cronbach's alpha values for the overall scale and each subscale of the IPERSS. The factors identified through the EFA were considered subscales within the overall scale. Each subscale score was calculated by summing the included items.

Predictors of the scores on the IPERSS

Correlations between the overall scale and each subscale's score on the IPERSS, overall score on the NES, and each subscale's score on the EssenCES-JPN were computed. Univariate analyses, including unpaired t-tests, and one-way analyses of variance and post hoc analyses (Bonferroni correction) were conducted on the overall scale and each subscale of the IPERSS.

Forward-backward stepwise multiple regression analyses were conducted to control for confounding variables (inclusion value = .05 and exclusion value = .10). In the stepwise multiple regression analyses, the overall scale, and each subscale scores on the IPERSS were entered as dependent variables. The variables for which the correlation analyses and univariate analyses yielded significant results were entered as independent variables.

Ethical considerations

Ethical approval to conduct this study was granted by the ethics committee of the Graduate School of Medicine, Nagoya University, Japan (No: 17-155). The participants were informed about the aims of the study and the benefits and risks of participation through printed forms. The participants provided written informed consent. They responded to each questionnaire anonymously, enclosed the completed questionnaires in sealed envelopes, and returned them to the researchers. Hospital directors and nurse managers did not participate in

the data collection process. If participants had any questions about the study, they were able to call or email the principal investigator, whose phone number and address were listed on the documents for research cooperation. Permissions to use the EssenCES-JPN and the NES were obtained from the copyright holders.

Results

Data were collected from 823 RNs (response rate = 41.25%). Excluding missing responses, the final sample size was 724 (valid response rate = 36.29%). The demographic characteristics of the participants and the features of the hospitals/wards in which they worked are presented in Table 1.

Table 1. Characteristics of the participants and features of the hospitals/wards in which they were working (n = 724)

		n	%
**Participants**	**Gender**		
	Male	324	44.8
	Female	400	55.2
	**Years of psychiatric experience**		
	< 5	191	26.4
	5 – 14	323	44.6
	≥ 15	210	29.0
	**APRN status[a]**		
	No	698	96.4
	Yes	26	3.6
	**Experience as a psychiatric home visiting nurse**		
	No	555	76.7
	Yes	169	23.3

	**Experience in providing psychiatric outpatient care**		
	No	652	90.1
	Yes	72	9.9
	**Experience in somatic care wards**		
	No	264	36.5
	Yes	460	63.5
	**Educational level**		
	Diploma	598	82.6
	Bachelor	112	15.5
	Master	14	1.9
**Hospitals/ Wards**	**Hospital establishment**		
	Public	366	50.6
	Non-profit corporation	27	3.7
	Private	331	45.7
	**Adoption of primary nursing**		
	No	325	44.9
	Yes	399	55.1
	**Holding pre-discharge conferences usually**		
	No	166	2.9
	Yes	558	77.1
	**Participation of families in predischarge conferences**		
	No	122	16.9
	Yes	436	60.2
	**Participation of multidisciplinary teams in predischarge conferences**		
	Consist only of hospital staff	312	43.1
	Consist of both community and hospital staff	246	34.0

[a] Certified nurse or certified nurse specialist

Latent structure of the IPERSS

No ceiling or floor effects were observed for any of the items of the IPERSS. Based on the results of inter-item correlation analyses, Item 3 was excluded because its coefficients with all items were lower than .30. None of the item-total correlations of the items were lower than .30. The descriptive statistics of the IPERSS are shown in S1 Table.

In the EFA, six items (Items 1, 2, 19, 20, 27, and 36) were excluded because their loadings onto all factors were lower than .40 or cross-loaded (loadings > .40) onto two or more factors. The final model of the IPERSS was a 5-factor structure that consisted of 36 items. Further, the KMO statistic was .96, and Bartlett's test of sphericity yielded significant results (χ^2 = 14 772, df = 630, p < .01). The collected data were considered superior [32]. Table 2 presents the results of the EFA of the 36-item IPERSS. Factor 1 was labeled "Promoting cognitive functioning and self-care" and consisted of nine items. Factor 2 was labeled "Identifying reasons for readmission" and consisted of eight items. Factor 3 was labeled "Establishing cooperative systems within the community" and consisted of seven items. Factor 4 was labeled "Sharing goals about community life" and consisted of seven items. Factor 5 was labeled "Creating restful spaces" and consisted of five items. Their Cronbach's alphas ranged from .81 to .91 (see Table 2). The mean overall score of the IPERSS was 131.92 (SD = 18.09). The Cronbach's alpha of the 36-item IPERSS was .96.

Table 2. Exploratory factor analyses and internal consistency of the IPERSS (n = 724)

**Total: IPERSS** (α = .958)		Factor loadings					$h^{2\,a}$
		F1	F2	F3	F4	F5	
**Factor 1: Promoting cognitive functioning and self-care** (α = .908)							
#30	I talked with the patient about how to deal with delusions so that he/she could take responsibility for behaviors.	**.960**	-.072	-.075	.035	-.137	.648
#29	I helped the patient reconsider his/her thoughts so that he/she	**.926**	-.071	-.047	.048	-.183	.591

	could realize that his/her delusions were thoughts that were inconsistent with reality.						
#33	I observed how the patient deals with delusions.	**.741**	-.012	-.013	-.024	.057	.564
#31	I evaluated my nursing care from changes in the patient's behaviors.	**.676**	.071	.055	-.025	-.037	.514
#32	I believed in the patient and encourage him/her to change behaviors.	**.592**	.126	.000	-.056	.088	.503
#34	I helped the patient understand the need for medication by providing factual information.	**.581**	-.002	.067	.012	.122	.524
#35	I tried to notice changes in the patient's attitudes toward medication	**.554**	.119	.087	-.051	.101	.544
#28	I helped the patient improve his/her lifestyle.	**.493**	-.001	.073	.190	.064	.540
#26	I helped the patient accept his/her disability.	**.427**	.120	.052	.019	.137	.453
	**Factor 2: Identifying reasons for readmission** (α = .883)						
#9	I tried to understand the patient's capabilities and the challenges that he/she had been facing.	-.025	**.828**	.036	.028	-.104	.616
#8	I tried to gain a more detailed understanding about why the patient was readmitted.	-.036	**.738**	.029	-.022	-.001	.510
#7	I tried to understand the challenges that the patient had been facing based on my observations of his/her daily life.	.139	**.723**	-.024	-.046	-.058	.532
#6	As a nurse, I paid close attention to the things about which the patient was worried.	.085	**.718**	-.050	-.106	.075	.525
#10	I envisioned nursing goals that aimed to enhance the post-discharge well-being of the patient.	-.034	**.660**	.019	.268	-.113	.623
#11	I created nursing plans that incorporated the opinions of other nursing staff members.	-.051	**.578**	.039	.171	.010	.508
#5	I shared important information about the patient with other nursing staff members.	-.035	**.536**	-.055	-.064	.223	.371
#4	I often cooperated with other nursing staff members.	-.048	**.430**	.052	.163	.047	.359

**Factor 3: Establishing cooperative systems within the community** (α = .882)							
#42	I discussed with the patient the services that he/she wanted to use in his/her community.	.077	-.074	**.845**	-.044	-.073	.629
#40	I participated in care conferences that involved community care providers.	-.140	.032	**.844**	.064	-.096	.606
#43	I informed the patient and his/her family about the support system that would be available to the patient after discharge.	.068	-.019	**.760**	-.097	-.101	.654
#41	I assessed the patient’s self-care abilities by comparing his/her behaviors at the time of admission and discharge.	.127	.040	**.734**	-.122	-.038	.548
#38	I put the patient in touch with community nurses to ensure the continuity of care.	-.155	.042	**.685**	.032	.129	.503
#37	I was in touch with the patient and his/her caregivers, for a while, even after discharge.	.021	.002	**.497**	.062	.209	.486
#39	I discussed with the patient the good things that could happen to him/her after discharge with him/her.	.209	-.006	**.430**	-.038	.097	.382
**Factor 4: Sharing goals about community life** (α = .891)							
#15	I discussed with the patient and his/her family how he/she can adapt to community life.	-.068	.034	.051	**.840**	-.081	.648
#14	I discussed with the patient and his/her family what he/she wanted to do in his/her community.	.032	.000	.021	**.789**	-.096	.581
#17	I helped the patient prepare for community life.	.052	-.080	.059	**.699**	.087	.606
#16	I helped the patient practice what he/she was not good at.	.065	-.004	-.018	**.668**	.105	.573
#18	I reassured the patient and his/her family about his/her ability to adapt to community life after discharge.	.065	-.004	-.018	**.645**	.110	.571
#13	I relieved the anxiety that the patient and his/her family experienced about community life.	.096	.077	.031	**.550**	-.090	.407
#12	I valued the happiness of the patient and his/her family.	.044	.202	-.080	**.549**	-.005	.473
**Factor 5: Creating restful spaces** (α = .810)							
#23	I created spaces within which the patient did not feel stressed.	-.099	-.022	-.040	-.114	**.890**	.524

#22	I allowed the patient to take rest and calm himself/herself down.	.119	.049	-.119	.063	**.589**	.484
#24	I shared nursing goals with other nursing staff members.	-.071	.024	.073	.131	**.575**	.464
#21	I assured the patient that hospitals are safe spaces.	.156	.108	-.005	.086	**.438**	.498
#25	I helped the patient feel more hopeful about community life.	.180	.008	.051	.087	**.409**	.429
	Eigenvalues	14.8	2.2	2.0	1.2	1.2	
	Inter-factor correlation F1	1					
	F2	.644	1				
	F3	.632	.554	1			
	F4	.655	.749	.672	1		
	F5	.707	.697	.522	.677	1	

Abbreviations: IPERSS = In-hospital nursing care to prevent early readmission in patients with schizophrenia scale

[a] h^2: Communalities

Predictors of the scores on the IPERSS

The mean scores of the overall NES, “Patients’ cohesion and mutual support”, “Experienced safety”, and “Therapeutic hold” were 121.65 (SD = 18.42), 10.06 (SD = 2.57), 6.48 (SD = 3.82), and 12.58 (SD = 2.93), respectively. Table 3 shows the correlation matrix for the overall scale and each subscale’s score on the IPERSS, each subscale’s score on the EssenCES-JPN, and the overall score on the NES. The correlation obtained between the overall score on the IPERSS and the overall score on the NES was $r = .59$, $p < .01$, between the overall score on the IPERSS and the score on the “Patients’ cohesion and mutual support” was $r = .18$, $p < .01$; between the overall score on the IPERSS and the score on the “Experienced safety” was $r = -.07$, $p = .06$; and between the overall score on the IPERSS and the score on the “Therapeutic hold” was $r = .33$, $p < .01$.

Table 3. Correlation between the overall scale and each subscale score on the IPERSS,

each subscale score of the EssenCES-JPN, and the overall score on the NES (n = 724)

Scales	NES	PC	ES	TH
**IPERSS total score**	.590**	.180**	-.071	.331**
**Factor 1**: Promoting cognitive functioning and self-care	.534**	.152**	-.095*	.260**
**Factor 2**: Identifying reasons for readmission	.547**	.119**	-.033	.315**
**Factor 3**: Establishing cooperative systems within the community	414**	.182**	-.076*	.277**
**Factor 4**: Sharing goals about community life	.472**	.160**	-.037	.257**
**Factor 5**: Creating restful spaces	.512**	.133**	-.039	.284**

Abbreviations: IPERSS = In-hospital nursing care to prevent early readmission in patients with schizophrenia scale, NES = Nursing excellence scale in clinical practice, PC = Patients' cohesion and mutual support, ES = Experienced safety, TH = Therapeutic hold

*p < .01, **p < .05

The results of the univariate analyses, namely, the unpaired t-tests, and one-way analyses of variance and the post hoc analyses (Bonferroni correction), on the overall scale score and each subscale's score on the IPERSS are summarized in Table 4. Significant differences were found in the univariate analyses with the overall score on the IPERSS as a dependent variable and the following nine variables as independent variables: years of psychiatric experience, APRN status, experience as a psychiatric home visiting nurse, experience in providing psychiatric outpatient care, educational level, hospital establishment, the adoption of primary nursing, the participation of families in pre-discharge conferences, and the participation of multidisciplinary teams in pre-discharge conferences.

Table 4. Univariate analyses of the IPERSS (n = 724)

		**Total** IPERSS		**Factor 1:** Promoting cognitive functioning and self-care		**Factor 2**: Identifying reasons for readmission		**Factor 3**: Establishing cooperative systems within the community		**Factor 4**: Sharing goals about community life		**Factor 5**: Creating restful spaces	
	n	Mean	(SD)	Mean	(SD)	Mean	(SD)	Mean	(SD)	Mean	(SD)	Mean	(SD)
**Gender**[a]													
Male	324	131.6	(17.7)	32.5	(5.1)	30.0	(4.4)	24.6	(4.6)	25.7	(4.1)	18.9	(2.6)
Female	400	132.2	(18.4)	32.2	(5.4)	30.6	(4.6)	24.7	(4.1)	25.7	(4.3)	18.9	(2.8)
		t = -.38		t = .67		t = -1.82		t = -.47		t = -.17		t = .29	
		p = .71		p = .50		p = .07		p = .64		p = .87		p = .78	
**Years of psychiatric experience**[b]													
< 5	191	128.1	(17.3)[c]	31.4	(5.0)[c]	29.6	(4.3)[c]	23.8	(4.9)[c]	24.9	(4.3)[c]	18.5	(2.6)[c]
5 – 14	323	131.1	(17.9)[d]	32.1	(5.4)[d]	30.2	(4.5)[f]	24.4	(4.7)[d]	25.6	(4.1)[f]	18.8	(2.7)
≥ 15	210	136.6	(18.2)[c, d]	33.5	(5.3)[c, d]	31.3	(4.5)[c, f]	25.9	(4.9)[c, d]	26.6	(4.0)[c, f]	19.4	(2.8)[c]
		F = 12.06		F = 8.32		F = 7.40		F = 10.45		F = 8.89		F = 6.34	
		p < .01		*p < .01*		*p < .01*		*p < .01*		*p < .01*		*p < .01*	
**APRN status**[a]													
No	698	131.4	(18.0)	32.2	(5.3)	30.2	(4.5)	24.6	(4.9)	25.6	(4.2)	18.8	(2.7)
Yes	26	145.9	(16.0)	35.4	(4.5)	33.5	(3.2)	27.3	(4.8)	28.9	(4.1)	20.9	(2.6)
		t = -4.06		t = -3.07		t = -3.68		t = -2.78		t = -3.94		t = -3.85	
		p < .01		*p < .01*		*p < .01*		*p < .01*		*p < .01*		*p < .01*	
**Experience as a psychiatric home visiting nurse**[a]													
No	555	130.5	(17.9)	32.0	(5.2)	30.2	(4.4)	24.2	(4.9)	25.5	(4.3)	18.8	(2.7)
Yes	169	136.5	(18.1)	33.5	(5.4)	30.9	(4.6)	26.2	(4.6)	26.5	(3.9)	19.3	(2.8)
		t = -3.76		t = -3.33		t = -1.88		t = -4.73		t = -2.93		t = -2.39	
		p < .01		*p < .01*		p = .06		*p < .01*		*p < .01*		*p = .02*	
**Experience in providing psychiatric outpatient care**[a]													

No	652	131.4	(18.0)	32.3	(5.3)	30.2	(4.5)	24.5	(4.9)	25.6	(4.2)	18.9	(2.7)
Yes	72	136.2	(18.0)	32.9	(5.6)	31.9	(4.2)	25.7	(4.7)	26.5	(4.3)	19.2	(2.8)
		t = -2.12		t = -1.02		t = -3.20		t = -1.95		t = -1.62		t = -.86	
		p = .03		p = .31		*p < .01*		p = .05		p = .11		p = .39	
**Experience in somatic care wards[a]**													
No	264	132.0	(17.7)	32.5	(5.0)	30.5	(4.1)	24.6	(4.8)	25.7	(4.3)	18.8	(2.7)
Yes	460	131.9	(18.3)	32.2	(5.5)	30.3	(4.7)	24.7	(4.9)	25.7	(4.1)	18.9	(2.7)
		t = .13		t = .85		t = -.55		t = -.38		t = -.25		t = -.63	
		p = .90		p = .40		p = .58		p = .70		p = .80		p = .53	
**Education level[b]**													
Diploma	598	131.4	(18.1)[f]	32.2	(5.4)[f]	30.2	(4.5)	24.6	(4.9)	25.6	(4.1)	18.8	(2.7)[f]
Bachelor	112	133.3	(17.5)	32.6	(4.7)	30.7	(4.5)	24.8	(4.8)	26.1	(4.6)	19.2	(2.8)
Master	14	143.6	(19.0)[f]	35.9	(5.1)[f]	32.6	(4.2)	26.9	(4.9)	27.7	(4.8)	20.6	(2.8)[f]
		F = 3.57		F = 3.47		F = 2.50		F = 1.50		F = 2.25		F = 3.86	
		p = .03		*p = .03*		p = .08		p = .22		p = .11		*p = .02*	
**Hospital establishment[b]**													
Public	366	134.3	(17.5)[c]	32.9	(5.3)[c]	30.9	(4.2)[c]	24.9	(4.9)	26.2	(4.1)[c]	19.3	(2.7)[c]
Non-profit corporation	27	129.8	(19.1)	31.5	(6.0)	29.6	(3.9)	24.6	(5.0)	25.6	(4.6)	18.5	(2.7)
Private	331	129.5	(18.3)[c]	31.7	(5.1)[c]	29.8	(4.7)[c]	24.4	(4.9)	25.1	(4.2)[c]	18.5	(2.7)[c]
		F = 6.57		F = 4.96		F = 6.54		F = 1.26		F = 6.17		F = 7.38	
		p < .01		*p < .01*		*p < .01*		p = .28		*p < .01*		*p < .01*	
**Adoption of primary nursing[a]**													
No	325	128.8	(17.8)	31.6	(5.2)	29.7	(4.7)	23.9	(4.9)	25.1	(4.1)	18.5	(2.7)
Yes	399	134.4	(17.9)	32.9	(5.3)	30.9	(4.3)	25.3	(4.8)	26.2	(4.3)	19.2	(2.7)
		t = -4.21		t = -3.14		t = -3.81		t = -3.69		t = -3.65		t = -3.23	
		p < .01		*p < .01*		*p < .01*		*p < .01*		*p < .01*		*p < .01*	
**Participation of families in pre-discharge conferences[b]**													

Not hold pre-discharge conferences	166	128.1	(18.3)[c]	32.1	(5.3)	29.6	(4.8)[f]	23.0	(5.1)	24.8	(4.5)[c]	18.6	(2.8)
No	122	129.2	(19.5)[f]	32.1	(5.9)	29.9	(4.7)	23.7	(5.0)[d]	24.7	(4.4)[d]	18.7	(2.8)
Yes	436	134.1	(17.3)[c, f]	32.5	(5.1)	30.8	(4.2)[f]	25.6	(4.5)[c, d]	26.3	(3.9)[c, d]	19.0	(2.7)
		F = 8.55		F = .461		F = 4.82		F = 20.07		F = 12.37		F = 1.54	
		p < .01		p = .63		*p < .01*		*p < .01*		*p < .01*		p = .22	
**Participation of multidisciplinary in pre-discharge conferences[b]**													
Not hold pre-discharge conferences	166	128.1	(18.3)[c]	32.1	(5.3)	29.6	(4.8)[c, d]	23.0	(5.1)[c, d]	24.8	(4.5)[c]	18.6	(2.8)
Only hospital staff	312	131.3	(17.7)[f]	32.2	(5.4)	30.2	(4.2)[c e]	24.4	(4.7)[c e]	25.6	(4.0)	19.0	(2.7)
Community and hospital staff members	246	135.2	(18.0)[c, f]	32.7	(5.2)	31.1	(4.5)[d, e]	26.1	(4.5)[d, e]	26.4	(4.1)[c]	19.0	(2.7)
		F = 8.14		F = .85		F = 6.14		F = 21.83		F = 7.31		F = .98	
		p < .01		p = .43		*p < .01*		*p < .01*		*p < .01*		p = .38	

Abbreviations: IPERSS = In-hospital nursing care to prevent early readmission in patients with schizophrenia scale, SD = standard deviation, APRN = advanced practice registered nurse (certified nurse or certified nurse specialist)

[a]: unpaired t-tests

[b]: one-way analyses of variance, and post-hoc analyses (Bonferroni correction)

[c, d, e] $p < .01$ (a significant difference was observed between groups)

[f, g] $p < .05$ (a significant difference was observed between groups)

In a stepwise multiple regression analysis with the overall score on the IPERSS as the dependent variable, the following 12 variables were entered as independent variables: years of psychiatric experience, APRN status, experience as a psychiatric home visiting nurse,

experience in providing psychiatric outpatient care, educational level, hospital establishment, the adoption of primary nursing, the participation of families in pre-discharge conferences, the participation of multidisciplinary teams in pre-discharge conferences, the score on “Patients’ cohesion and mutual support”, the score on “Therapeutic hold”, and the overall score on the NES. The stepwise multiple regression analyses revealed that the overall scale score on the IPERSS was predicted by the following five variables: the overall score on the NES (β = .53, p < .01), the score on “Therapeutic hold” (β = .12, p < .01), APRN status (β = .09, p < .01), the participation of multidisciplinary teams in pre-discharge conferences (β = .08, p = .01) , and the participation of families in pre-discharge conferences (β = .07, p = .03). The results of the multiple stepwise regression analyses are presented in Table 5.

Table 5: Stepwise multiple regressions of the IPERSS (n = 724)

	**Total** IPERSS		**Factor 1**: Promoting cognitive functioning and self-care		**Factor 2**: Identifying reasons for readmission		**Factor 3**: Establishing cooperative systems within the community		**Factor 4**: Sharing goals about community life		**Factor 5**: Creating restful spaces	
	B [95% CI]	*β*	B [95% CI]	*β*	B [95% CI]	*β*	B [95% CI]	*β*	B [95% CI]	*β*	B [95% CI]	*β*
**APRN status[a]**												
No	Reference				Reference				Reference		Reference	
Yes	8.36 [2.76, 13.96]	*.09***			1.85 [.40, 3.30]	*.08**			2.10 [.68, 3.53]	*.09***	1.27 [.36, 2.18]	*.09***
	VIF = 1.01				VIF = 1.01				VIF = 1.01		VIF = 1.01	
**Experience as a psychiatric home visiting nurse**												
No							Reference					
Yes							1.26 [.52, 2.00]	*.11***				
							VIF = 1.03					
**Education level**												

Diploma		Reference				
Bachelor						
Master		.15 [.03, .27] *.08**<br>VIF = 1.03				
**Participation of families in pre-discharge conferences**						
Not hold the conferences	Reference			Reference	Reference	
No						
Yes	2.61 [.25, 4.97] *.07**<br>VIF = 1.24			1.28 [.58, 1.98] *.13***<br>VIF = 1.25	1.34 [.80, 1.88] *.16***<br>VIF = 1.00	
**Participation of multidisciplinary teams in pre-discharge conferences**						
Not hold the conferences	Reference		Reference	Reference		
Only hospital staff						
Community and hospital staff members	3.16 [.68, 5.46] *.08**<br>VIF = 1.24		.96 [.39, 1.53] *.10***<br>VIF = 1.01	1.41 [.69, 2.14] *.14***<br>VIF = 1.24		
**NES**	.52 [.46, .58] *.53***<br>VIF = 1.16	.14 [.13, .16] *.50***<br>VIF = 1.15	.12 [.10, .14] *.49***<br>VIF = 1.16	.09 [.07, .11] *.34***<br>VIF = 1.17	.10 [.09, .12] *.44***<br>VIF = 1.04	.07 [.06, .08] *.46***<br>VIF = 1.16
**PC**				.16 [.03, .28] *.08**<br>VIF = 1.11	.14 [.03, .24] *.08**<br>VIF = 1.03	
**ES**		-.122 [-.21, -04] *-.09***<br>VIF = 1.00				
**TH**	.77 [.39, 1.15] *.12***<br>VIF = 1.16	.15 [.03, .27] *.08**<br>VIF = 1.16	.20 [.10, .30] *.13***<br>VIF = 1.16	.17 [.05, .28] *.10***<br>VIF = 1.26		.11 [.05, .17] *.12***<br>VIF = 1.14
R^2	.389	.303	.332	.262	.263	.282

Adjusted R^2	.385	.299	.328	.256	.259	.279

Abbreviations: IPERSS = In-hospital nursing care to prevent early readmission in patients with schizophrenia scale, CI = confidence interval, APRN = advanced practice registered nurse (certified nurse or certified nurse specialist), NES = Nursing excellence scale in clinical practice, PC = Patients' cohesion and mutual support, ES = Experienced safety, TH = Therapeutic hold

$^{*}p < .01$, $^{**}p < .05$

Discussion

The main findings of this study can be summarized as follows: the IPERSS was found to consist of five factors and 36 items, and the overall scores of the IPERSS were significantly associated with APRN status, the overall score on the NES, the score on "Therapeutic hold", the participation of families in pre-discharge conferences, and the participation of multidisciplinary teams in pre-discharge conferences. To the best of our knowledge, this is the first study to investigate the latent structure and predictors of in-hospital nursing care to prevent early readmissions in patients with schizophrenia.

Latent structure of the IPERSS

The item analyses made it possible to avoid collinearity of items in the IPERSS and ensure sufficient internal consistency. Item 3, which was excluded in the inter-item correlation analysis on the 43-item IPERSS, was not included in "In-hospital nursing care to prevent early readmission", because the correlation coefficients between Item 3 and all the other items were less than .30.

The five-factor structure of the IPERSS was largely consistent with the findings of the previous study [34]. The previous study revealed qualitatively that nursing practices by hospital nurses to prevent the readmission in patients with schizophrenia could be classified into five

categories. Each factor of the IPERSS largely overlapped with each of Maki et al.'s five categories as follows [34]: Factor 1 of the IPERSS (i.e., Promoting cognitive functioning and self-care), Category 1 in the previous study (i.e., Supporting the patient so that he/she can accept his/her illness); Factor 2 (i.e., Identifying reasons for readmission), Category 2 (i.e., As a sympathizer, understanding the patient's tasks and predicting the post-discharge life of each patient); Factor 3 (i.e., Establishing cooperative systems within the community), Category 3 (i.e., Bridging the gap between the community and the hospital, including nursing in the hospital, as part of the process); Factor 4 (i.e., Sharing goals about community life), Category 4 (i.e., Mediating between the patient and the family, with consideration for the happiness of both the patient and his/her family after discharge); Factor 5 (i.e., Creating restful spaces), Category 5 (i.e., Establishing a relationship of trust with the patient, ensuring the patient's sense of security, and considering his/her impaired ability to establish rapport). This study showed that Japanese nurses working in hospitals provided nursing care consisting of these five factors to prevent early readmission in patients with schizophrenia.

The five factors of the IPERSS include several elements that may influence the risk of readmission. Most studies reported that the existence or the number of previous admissions was associated with the risk of readmission [12]. Factor 2 of the IPERSS (i.e., Identifying reasons for readmission) can be useful for finding the reasons for patients' past hospitalizations and can be linked to the care to prevent early readmission. For example, Item 9 of the IPERSS (i.e., I tried to understand the patient's capabilities and the challenges that he/she had been facing), and Item 8 of the IPERSS (i.e., I tried to gain a more detailed understanding about why the patient was readmitted), could help to identify reasons for past hospitalizations. Self-management education interventions were associated with a significant reduction of re-hospitalization and more likely to improve patients' adherence to medication [45]. Factor 1 of the IPERSS (i.e., Promoting cognitive functioning and self-care) had much in common with

self-management education interventions. In self-management education, patients learn problem solving skills which allow them to take appropriate actions to improve their health [46]. Item 30 of the IPERSS (i.e., I talked with the patient about how to deal with delusions so that he/she could take responsibility for behaviors), and Item 29 of the IPERSS (i.e., I helped the patient reconsider his/her thoughts so that he/she could realize that his/her delusions were thoughts that were inconsistent with reality) may be effective for patients to acquire behaviors to improve their health.

Predictors of the scores on the IPERSS

The score on the NES

The score on the NES in this study may reflect the excellence of psychiatric hospital nurses all over Japan. The mean score on the overall NES in this study was similar to that of the previous study [42]. In the previous study, a survey using the NES was performed on 799 nurses in Japan, and the average score on the overall NES was 122.0 (SD = 18.1). This study also conducted a survey of hospital nurses across Japan. The excellence of the participants in this study might reflect that of nurses all over Japan.

The score on the overall NES may represent nurses' personal factors in their nursing performance indicated by the score on the overall IPERSS. The NES consisted of seven subscales. Each of these subscales was a personal component of nursing excellence. Nursing performance was influenced by not only individual factors but also environmental factors [30, 41]. In this study’s stepwise regression analysis, the score on the overall NES was the most significant variable among predictor variables. The individual factors of nurses, which was represented by the score on the overall NES, might predict most of the scores on the overall IPERSS.

Therapeutic ward climate

In Japan, nurses' perception of the social climate may be less therapeutic than the social climate perceived by nurses in European countries. The mean score on "Therapeutic hold" in this study was lower than in closed general psychiatric wards in Germany (i.e., mean = 13.8, SD was not reported) [36], lower than in open general psychiatric wards in Germany (i.e., mean = 15.6, SD was not reported) [36], and lower than in English high-security hospital settings (i.e., mean = 14.17, SD = 3.29) [47]. The average score on "Therapeutic hold" in this study was similar to that of a previous study in Japan (i.e., mean = 13.44, SD = 3.38) [40]. Japanese nurses may perceive the social climate in psychiatric wards to be less sensitive of patients' needs than foreign nurses.

The therapeutic ward climate was confirmed to be among the predictors of in-hospital nursing care to prevent early readmission. Nursing performance is influenced by nurses' personal, environmental, and patients' factors [48]. The R-squared in the stepwise multiple regression analyses of this study was not high enough. This was probably because the variables related to patients' characteristics were not entered in the stepwise regression analyses. If the variables on patients' characteristics were entered into the stepwise regression analyses, the R-squared may have been higher. However, no data related to patients' characteristics were obtained in this study. This study could reveal only that environmental factors as well as nurses' individual factors influenced in-hospital nursing care to prevent early readmission in patients with schizophrenia.

Systems that facilitate communication with community care providers

The present findings underscore the need to develop systems that facilitate communication between patients and community care providers such as family and multidisciplinary teams. The results of the multiple regression analyses revealed that the score on the overall IPERSS was significantly associated with the participation of families and

multidisciplinary teams (both community and hospital staff members) in pre-discharge conferences. To the best of our knowledge, no existing intervention facilitates communication between patients and community care providers immediately after readmission; therefore, such an intervention should be developed.

Limitations

This study has several limitations. First, the response rate in this study was low. The generalizability of our findings may be limited. The results of this study may have been influenced by the answers of nurses who had high standards of nursing excellence and were supportive of patients' needs. Second, nurses' responses may have been influenced by recall biases. The participants may have answered desirably without total recall. The scales used in this study could have been better evaluated. Third, this study did not investigate the data of patients, therapies that patients were offered, and the duration of hospitalization. The nursing care provided to patients depended on their mental state and social background. It is necessary to reveal the degree that the patients' characteristics influenced nursing care to prevent early readmission.

Conclusion

This study elucidated that Japanese nurses working in hospitals provide nursing care consisting of five factors to prevent early readmission in patients with schizophrenia. In-hospital nursing care to prevent early readmission in patients with schizophrenia was associated with not only nurses' individual factors but also environmental factors such as therapeutic ward climate, the participation of families in pre-discharge conferences, and the participation of multidisciplinary teams in pre-discharge conferences. To facilitate in-hospital nursing care aimed at preventing early readmission nurses should foster a social climate in which patients

feel cared for and develop systems that facilitate communication with community care providers.

Acknowledgments

We would like to thank the nurses who participated in this study, as well as the graduate students, the university faculty members, the psychiatric nurses, and the psychiatrist for participating in discussions on this research.

References

1. Pillai A, Schooler NR, Peter D, Looney SW, Goff DC, Kopelowicz A, et al. Predicting relapse in schizophrenia: Is BDNF a plausible biological marker? Schizophrenia research. 2018;193: 263-268.
2. Marder SR, Cannon TD. Schizophrenia. The New England journal of medicine. 2019;381(18): 1753-1761.
3. Chi MH, Hsiao CY, Chen KC, Lee LT, Tsai HC, Hui Lee I, et al. The readmission rate and medical cost of patients with schizophrenia after first hospitalization - A 10-year follow-up population-based study. Schizophrenia research. 2016;170(1): 184-190.
4. Donisi V, Tedeschi F, Salazzari D, Amaddeo F. Pre- and post-discharge factors influencing early readmission to acute psychiatric wards: implications for quality-of-care indicators in psychiatry. General hospital psychiatry. 2016;39: 53-58.
5. Joynt KE, Jha AK. Thirty-day readmissions — truth and consequences. The New England journal of medicine. 2012;366: 1366-1369.
6. Vigod SN, Kurdyak PA, Seitz D, Herrmann N, Fung K, Lin E, et al. READMIT: a clinical risk index to predict 30-day readmission after discharge from acute psychiatric units. Journal of psychiatric research. 2015;61: 205-213.

7. Han KT, Lee SY, Kim SJ, Hahm MI, Jang SI, Kim SJ, et al. Readmission rates of South Korean psychiatric inpatients by inpatient volumes per psychiatrist. BMC psychiatry. 2016;16:96. doi: 10.1186/s12888-016-0804-y.
8. Lay B, Kawohl W, Rossler W. Predictors of compulsory re-admission to psychiatric inpatient care. Frontiers in psychiatry. 2019;10:120. doi: 10.3389/fpsyt.2019.00120.
9. Lorine K, Goenjian H, Kim S, Steinberg AM, Schmidt K, Goenjian AK. Risk factors associated with psychiatric readmission. The Journal of nervous and mental disease. 2015;203(6): 425-430.
10. Vigod SN, Kurdyak PA, Dennis CL, Leszcz T, Taylor VH, Blumberger DM, et al. Transitional interventions to reduce early psychiatric readmissions in adults: systematic review. The British journal of psychiatry. 2013;202(3): 187-194. doi:10.1192/bjp.bp.112.115030.
11. Hung YY, Chan HY, Pan YJ. Risk factors for readmission in schizophrenia patients following involuntary admission. PLOS ONE. 2017;12(10): e0186768.
12. Donisi V, Tedeschi F, Wahlbeck K, Haaramo P, Amaddeo F. Pre-discharge factors predicting readmissions of psychiatric patients: a systematic review of the literature. BMC psychiatry. 2016;16(1): 449. doi: 10.1186/s12888-016-1114-0.
13. Han KT, Kim SJ, Jang SI, Hahm MI, Kim SJ, Lee SY, et al. The outcomes of psychiatric inpatients by proportion of experienced psychiatrists and nurse staffing in hospital: New findings on improving the quality of mental health care in South Korea. Psychiatry research. 2015;229(3): 880-886.
14. Andrews CE, Baker K, Howell CJ, Cuerdo A, Roberts JA, Chaudhary A, et al. Risk of hospitalization due to medication nonadherence identified through EMRs of patients with psychosis. Psychiatric services. 2017;68(8): 847-850.

15. Chien WT, Mui J, Gray R, Cheung E. Adherence therapy versus routine psychiatric care for people with schizophrenia spectrum disorders: a randomised controlled trial. BMC psychiatry. 2016;16: 42. doi: 10.1186/s12888-016-0744-6.

16. Sariah AE, Outwater AH, Malima KI. Risk and protective factors for relapse among individuals with schizophrenia: a qualitative study in Dar es Salaam, Tanzania. BMC psychiatry. 2014;14: 240. doi: 10.1186/s12888-014-0240-9.

17. Sfetcu R, Musat S, Haaramo P, Ciutan M, Scintee G, Vladescu C, et al. Overview of post-discharge predictors for psychiatric re-hospitalisations: a systematic review of the literature. BMC psychiatry. 2017;17(1): 227. doi: 10.1186/s12888-017-1386-z.

18. Khaleghparast S, Ghanbari B, Kahani S, Malakouti K, SeyedAlinaghi S, Sudhinaraset M. The effectiveness of discharge planning on the knowledge, clinical symptoms and hospitalisation frequency of persons with schizophrenia: a longitudinal study in two hospitals in Tehran, Iran. Journal of clinical nursing. 2014;23(15-16): 2215-2221.

19. Vigod SN, Taylor VH, Fung K, Kurdyak PA. Within-hospital readmission: an indicator of readmission after discharge from psychiatric hospitalization. Canadian journal of psychiatry. 2013;58(8): 476-481.

20. Forchuk C, Martin ML, Chan YL, Jensen E. Therapeutic relationships: from psychiatric hospital to community. Journal of psychiatric and mental health nursing. 2005;12(5): 556-564.

21. Meehan J, Kapur N, Hunt IM, Turnbull P, Robinson J, Bickley H, et al. Suicide in mental health in-patients and within 3 months of discharge: National clinical survey. The British journal of psychiatry. 2006;188(FEB.): 129-134.

22. Walter F, Carr MJ, Mok PLH, Antonsen S, Pedersen CB, Appleby L, et al. Multiple adverse outcomes following first discharge from inpatient psychiatric care: a national cohort study. The Lancet. Psychiatry. 2019;6(7): 582-589.
23. Word Health Organization. WHO. Mental health atlas 2017. Geneva, Switzerland : World Health Organization; 2018.
24. Yamaguchi S, Ojio Y, Koike J, Matsunaga A, Ogawa M, Tachimori H, et al. Associations between readmission and patient-reported measures in acute psychiatric inpatients: a study protocol for a multicenter prospective longitudinal study (the ePOP-J study). International journal of mental health systems. 2019;13: 40. doi: 10.1186/s13033-019-0298-3.
25. Okumura Y, Sugiyama N, Noda T, Tachimori H. Psychiatric admissions and length of stay during fiscal years 2014 and 2015 in Japan: A retrospective cohort study using a nationwide claims database. Journal of epidemiology. 2019;29(8): 288-294.
26. Organization for Economic Co-operation and Development. OECD health statistics 2020; 2020 [cited Jul 18, 2020]. Database: figshare [Internet]. Available from: https://www.oecd.org/els/health-systems/health-data.htm.
27. Kasai K, Ando S, Kanehara A, Kumakura Y, Kondo S, Fukuda M, et al. Strengthening community mental health services in Japan. The Lancet. Psychiatry. 2017;4(4): 268-270.
28. Department of Mental Health Policy, National Institute of Mental Health, Japan. New Mental Health Welfare Document 2017; 2018 [cited May 14,2019]. Database: figshare [Internet]. Availale from: https://www.ncnp.go.jp/nimh/seisaku/data/. Japanese.

29. Cha C, Sung C. Factors related to nursing performance in South Korean intensive care units. International journal of nursing practice. 2020. e12874. https://doi.org/10.1111/ijn.12874.
30. Long CG, Anagnostakis K, Fox E, Silaule P, Somers J, West R, et al. Social climate along the pathway of care in women's secure mental health service: variation with level of security, patient motivation, therapeutic alliance and level of disturbance. Criminal behavior and mental health. 2011;21(3): 202-214.
31. Moos RH, Schaefer JA. Evaluating health care work settings: A holistic conceptual framework. Psychology & health. 1987;1(2): 97-122.
32. Pett M, Lackey N, Sullivan J. Making Sence of Factor Analysis. Califonia, USA: Sage Publications, Inc; 2003.
33. Ministry of Health, Labour and Welfare. Matters concerning medical treatment fee score table. 2020 March 5 [cited August 29, 2020]. In: web sites [Internet]. Available from: https://www.mhlw.go.jp/content/12400000/000603981.pdf. Japanese.
34. Maki S, Nagai K, Ando S. The process of the support by the proficient and expert hospital nurses for the community settlement of the discharged schizophrenia patients who were readmitted within three months. Journal of Japan society of nursing research. 2018;41(4): 713-722. doi: 10.15065/jjsnr.20180308015. Japanese.
35. Schalast N, Tonkin M. The Essen Climate Evaluation Schema - a manual and more. Göttingen, Germany: Hogrefe; 2016.
36. Siess J, Schalast N. Psychometric properties of the Essen Climate Evaluation Schema (EssenCES) in a sample of general psychiatric wards. Archieves of psychiatric nursing. 2017;31(6): 582-587.

37. Tonkin M, Howells K, Ferguson E, Clark A, Newberry M, Schalast N. Lost in translation? Psychometric properties and construct validity of the English Essen Climate Evaluation Schema (EssenCES) social climate questionnaire. Psychological assessment. 2012;24(3): 573-580.
38. Schalast N, Redies M, Collins M, Stacey J, Howells K. EssenCES, a short questionnaire for assessing the social climate of forensic psychiatric wards. Criminal and behaviour mental health. 2008;18(1): 49-58.
39. Noda S, Sugiyama N, Matsumoto Y, Tsujiwaki K, Hasegawa T, Ito H. Psychometric properties of the Japanese version of the Essen Climate Evaluation Schema (EssenCES). Clinical psychiatry. 2012;54(2): 211-217. Japanese.
40. Noda S, Sato M, Sugiyama N, Yoshihama F, Ito H. Assessment of ward climate by patients and nurse in psychiatric hospital using the Essen Climate Evaluation Schema: Japanese version (EssenCES-JPN). Clinical psychiatry. 2014;56(8): 715-722. Japanese.
41. Cho H, Han K. Associations among nursing work environment and health-promoting behaviors of nurses and nursing performance quality: A multilevel modeling approach. Journal of nursing scholarship. 2018;50(4): 403-410. doi: 10.1111/jnu.12390.
42. Kameoka T. Nursing Excellence Scale in Clinical Practice. In: Funashima N, editor. Measuring tool file for nursing practice and education. 3. Igakushoin: Tokyo, Japan; 2015. pp. 64-75. Japanese.
43. Kameoka T, Funashima N. Exploration of the attributes associated with excellence in nursing practice: Focused on nurses who have more than 5 years' clinical experience. The journal of nursing studies, national college of nursing, Japan. 2015;14(1): 1-10. Japanese.

44. Rozental A, Kottorp A, Boettcher J, Andersson G, Carlbring P. Negative effects of psychological treatments: An exploratory factor analysis of the negative effects questionnaire for monitoring and reporting adverse and unwanted events. PLOS ONE. 2016;11(6): e0157503.
45. Zou H, Li Z, Nolan MT, Arthur D, Wang H, Hu L. Self-management education interventions for persons with schizophrenia: a meta-analysis. International journal of mental health nursing. 2013;22(3): 256-271.
46. Bourbeau J, Julien M, Maltais F, Rouleau M, Beaupré A, Bégin R, et al. Reduction of hospital utilization in patients with chronic obstructive pulmonary disease: a disease-specific self-management intervention. Archives of internal medicine. 2003;163(5): 585-591.
47. Howells K, Tonkin M, Milburn C, Lewis J, Draycot S, Cordwell J, et al. The EssenCES measure of social climate: a preliminary validation and normative data in UK high secure hospital settings. Criminal and behaviour mental health. 2009;19(5): 308-320.
48. Campbell K, Massey D, Broadbent M, Clarke KA. Factors influencing clinical decision making used by mental health nurses to provide provisional diagnosis: A scoping review. International journal of mental health nursing. 2019;28(2): 407-424.

Supporting information

S1 Figure. In-hospital nursing care to prevent early readmissions

(DOCX)

S1 Table. Descriptive statistics of the IPERSS

(DOCX)

S1 Appendix. 43-item IPERSS (Engilsh).

(DOCX)

S2 Appendix. 43-item IPERSS (Japanese).

(DOCX)

S1 Dataset

(SAV)

Click here to access/download
Supporting Information
S1_Figure.docx

Supporting Information_1_Table

Click here to access/download
Supporting Information
S1_Table.docx

Click here to access/download
Supporting Information
S1_Appendix.docx

Click here to access/download
Supporting Information
S2_Appendix.docx

Supporting Information_1_Dataset

Click here to access/download

Supporting Information

S1_Dataset.sav
