# Supplementary material for: Structure and predictors of in-hospital nursing care leading to reduction in early readmission among patients with schizophrenia in Japan: A cross-sectional study
Source: PLoS One. 2021 Apr 30;16(4):e0250771. doi: 10.1371/journal.pone.0250771 (PMC8087037; doi:10.1371/journal.pone.0250771)
Supplement: Attachment — Submitted filename: Response to Reviewers.docx [file pone.0250771.s010.docx]

Dear Academic Editor and Reviewers

Thank you very much for reviewing our manuscript and offering valuable suggestions.

We have addressed your comments with point-by-point responses and revised the manuscript accordingly.

**Journal Requirements:**

Please review your reference list to ensure that it is complete and correct. If you have cited papers that have been retracted, please include the rationale for doing so in the manuscript text, or remove these references and replace them with relevant current references. Any changes to the reference list should be mentioned in the rebuttal letter that accompanies your revised manuscript. If you need to cite a retracted article, indicate the article’s retracted status in the References list and also include a citation and full reference for the retraction notice.

**Response**

Thank you for your comment. We have revised the Reference section. We have replaced the parts that should not be uppercase with lowercase and the parts that should not be lowercase with uppercase (Page 31, Line 512–Page 39, Line 719. in the revised manuscript).

We have cited no papers that have been retracted.

**Reviewer #2s’ comment:**

Abstract:

“About 15%–30% of patients with schizophrenia are readmitted within 90 days of discharge due to exacerbation of symptoms that leads to self-harm, harm to others, or self-neglect.” Is this data specific to Japan or from global research? Suggest specifying that this is referring to inpatient psychiatric admissions, e.g., “Globally, around 15-30% of patients with schizophrenia discharged from inpatient psychiatric admissions are readmitted within 90 days….”.

**Response**

Thank you very much for your valuable suggestion. As the reviewer states, we should specify that this data is from global research and that this sentence refers to inpatient psychiatric admissions. Therefore, we have revised this point as follows:

(Page 2. Line 26–28. Abstract)

“Globally, about 15%–30% of patients with schizophrenia discharged from inpatient psychiatric admissions are readmitted within 90 days due to exacerbation of symptoms that leads to self-harm, harm to others, or self-neglect.”

**Reviewer #2s’ comment:**

Discussion:

Line 410: “The R-squared in the stepwise multiple regression analyses of this study was not high enough” still suggests that there is a specific threshold for R-squared the authors were trying to reach. Suggest rewriting as “The R-squared in the stepwise multiple regression analyses of this study was not high” or specify a threshold.

**Response**

Thank you for your comment. As the reviewer states, “The R-squared in the stepwise multiple regression analyses of this study was not high enough” still suggests that there is a specific threshold for R-squared we were trying to reach. Therefore, we have deleted the word “enough” (Page 28, Line 451, and Line 456. in the original manuscript).
